# Assessment of cardiac variables using a new electrocardiography lead system in horses

**DOI:** 10.14202/vetworld.2020.1229-1233

**Published:** 2020-06-29

**Authors:** Worakij Cherdchutham, Kanoklada Koomgun, Suchanan Singtoniwet, Napattra Wongsutthawart, Napass Nontakanun, Wipasitnee Wanmad, Soontaree Petchdee

**Affiliations:** Department of Large Animal and Wildlife Clinical Sciences, Faculty of Veterinary Medicine, Kasetsart University, Kamphaeng Saen Campus 73140, Thailand

**Keywords:** echocardiography, electrocardiography, horse, non-invasive cardiac evaluation

## Abstract

**Aim:**

The objective of this study was to assess a new lead system method to improve electrocardiographic measurement in horses.

**Materials and Methods:**

Twenty-two horses with an average age of 8.8±0.8 years were enrolled in this study. Horses were divided into two groups, consisting of a control group (n=11) and athlete group (n=11). Electrocardiography (ECG) and echocardiography were performed to provide information on the structure and function of the heart. Two lead systems, base apex and modified precordial leads, were used for the electrocardiogram to assess the cardiac electrophysiological functions.

**Results:**

PR interval, QT interval, and QRS-T angle presented significant differences between the control and athlete groups when the modified precordial lead system was used. However, significant variations in the mean electrical axis were found when the base apex lead system was used. The modified precordial lead system resulted in more significant differences in cardiac electrophysiological parameters than the base apex lead system. In the athlete group, echocardiography showed cardiac adaptations such as increases in the left atrial and left ventricular dimensions and stroke volume and a decrease in heart rate in response to exercise and training. The observed differences in cardiac morphology and function between groups suggested differences in health performance in the athlete group.

**Conclusion:**

These data provided the first evidence that the modified precordial lead system improved statistical variation in ECG recording and provided the most reliable method for health screening in horses.

## Introduction

It has been widely shown that exercise training improves health outcomes, such as reducing the risk of cardiac diseases and has been shown to promote many health benefits [1]. However, sudden cardiac death and cardiovascular abnormalities are considered to occur frequently in both athlete and non-athlete populations [2-4]. In athlete horses, an unexplained sudden cardiac death has increased over the past 5 years [5]. Cardiovascular investigation is recommended to perform as an important component of health screening before participating in sport activities [6]. Electrocardiography (ECG) measurement is an important tool to assess the diagnosis of arrhythmias and is a gold standard for the clinical examination of electrical disturbances in horses [7,8]. Many studies have been reported to investigate the cardiac electrophysiological response in horses [9-13]. Equine ECG lead systems have been developed to obtain a clear ECG recording using different positions of the electrodes. Several lead systems were suggested to be useful for horses and other animals to diagnose cardiac electrophysiological problems [14]. However, there are few data available to evaluate the cardiac electrical function of the horse.

ECG and echocardiography measurements might be useful tools to assess the cardiac functions in the horses. In this present study, the precordial lead position on the horse was modified to verify the sensitivity of the detection of electrocardiographic variations in horses.

The objective of this study was to assess a new lead system method to improve electrocardiographic measurement in horses. We hypothesized that a modified precordial lead system can be used to determine a more appropriate method for cardiac electrophysiological property assessment in horses. The application of this new lead system method among horses might be contributed to improve the electrophysiological measurement of the horses.

## Materials and Methods

### Ethical approval

The study was approved by the Ethical Committee for Animal Experiments, Kasetsart University, Thailand (ACKU62-VET-061).

### Study location and period

The study performed at Ratchaburi Riding Club and Faculty of Veterinary Medicine, Kasetsart University, Kamphaeng Saen campus from March to June 2019.

### Animals

Six females and 16 males Thai native crossbred horses aged 8.8±0.8 years and weighing 304.7±18.2 kg were used for this study. The exclusion criteria were the presence of any health problems such as cardiac arrhythmias and structural heart diseases. Horses were assigned into two groups, the control group and the athlete group. All parameters were recorded continuously for further analysis using a blinded assessment.

### ECG measurements

**Figure-1:**
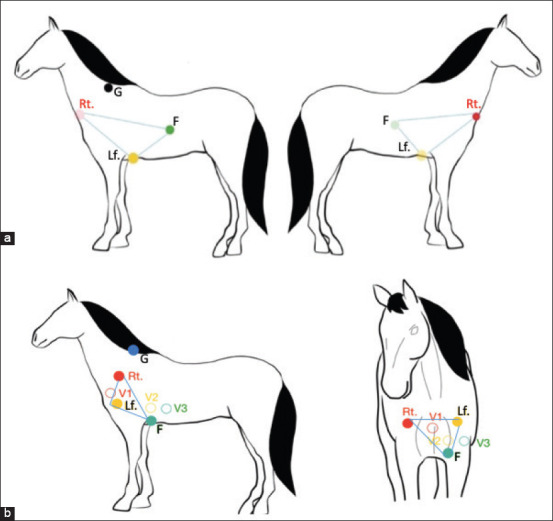
Two electrocardiography lead systems; base apex (a) and modified precordial leads (b), circle represented position of the electrodes; Rt, Lf, F, indicated locations of electrodes for the base apex leads and V1, V2, and V3 for the modified precordial leads, G represented ground electrode.

**Figure-2:**
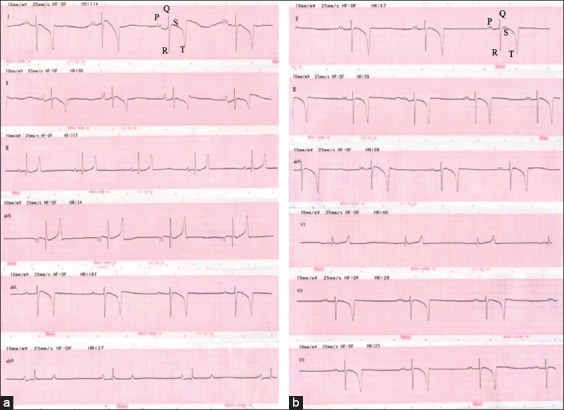
A section of base apex (a) and modified precordial lead (b), Electrocardiography signal such as P, Q, R, S, and T wave were obtained from the recording.

Non-invasive cardiac electrophysiological measurements were performed on 22 horses using a 12-channel ECG recording device (Kent, Japan). The ECGs were recorded at 25 mm/s paper speed with a sensitivity of 1 cm/mV. Six electrodes were placed on the skin with alligator clips to provide the 1 min-ECGs recordings without chemical restraint. Two ECG lead systems were used on each horse (Figure-1). A 1-min ECGs were recorded using each lead system (Figure-2), and the ECG parameters, such as P-wave duration and amplitude, PR interval, QRS duration and amplitude, QT interval, T-wave morphology, QRS-T angle, and mean electrical axis (MEA), were manually analyzed under the supervision of an experienced cardiologist. The heart MEA was calculated using Einthoven’s triangle as a reference. T-wave morphology was divided into three patterns; positive, negative, and biphasic.

### Cardiac function evaluations

**Figure-3:**
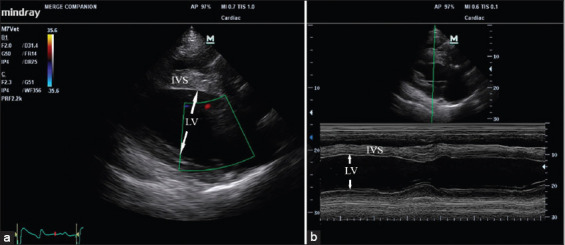
Echocardiographic of longitudinal image of heart (a) and M mode image of the left ventricle (b), arrow represented the left ventricular internal diameter; LV=left ventricle; IVS=Interventricular septum.

Transthoracic echocardiography was performed on resting horses to rule out the cardiac pathology and to evaluate the heart contractility functions. Echocardiographic examination was performed on an unsedated horse in standing position by one experienced cardiologist using a portable ultrasound system (Mindray, China). An echocardiogram with 4.0 MHz frequency transducer was used to obtain the images from the right and left sides of the thorax of the horse. Echocardiographic images were captured and stored for offline analysis. All measurements were the mean for three consecutive cardiac cycles. Left ventricular wall structure was calculated by measuring the images from standard two-dimensional plane and left ventricular systolic function was calculated from the M-mode echocardiography (Figure-3).

### Statistical analysis

All data are showed as mean ± standard error of the mean (mean ± SEM). Statistical analysis was performed using unpaired t-test (GraphPad Prism software version 5). p=0.05 or less was indicated for statistical significance. The coefficient of determination (R^2^) is calculated to analyze the variation of ECG parameters between the two leads system.

## Results

### General characteristic

All 22 horses (aged 8.8±0.8 years, and weighing 304.7±18.2 kg) completed the study without any complications. The characteristics of the study population are summarized in Table-1. There was no statistical difference between groups concerning mean age and body weight (p=0.11 and p=0.15, respectively). There were significant differences between the control and athlete horses in PR interval, QT interval, and QRS-T angle obtained by modified precordial leads and QT interval differences obtained by base apex lead. Several morphologies of the T wave were observed, and the biphasic configuration was generally presented in this study. The results of the analysis of ECG parameters are shown in Table-2. Table-3 represents a statistical measurement of ECG variations from the two lead systems. The variability in the MEA parameters was found in this study (Table-4).

**Table-1:** Characteristics of horses.

Horse characteristic	Control	Athlete
Gender	Female (6)	Female (1)
	Male (5)	Male (10)
Age (years)	10.4±1.6	7.5±0.7
Weight (kg)	265.0±23.7	309.5±17.7

**Table-2:** Electrocardiography measurements.

Lead system	Base apex		Modified precordial	
	
	Control	Athlete	Control	Athlete
P (ms)	0.11±0.01	0.14±0.01	0.10±0.01	0.13±0.01
P (mV)	0.22±0.02	0.20±0.02	0.20±0.02	0.21±0.02
PR (ms)	0.28±0.02	0.32±0.02	0.27±0.01*	0.32±0.02*
QRS (ms)	0.10±0.01	0.12±0.01	0.10±0.01	0.11±0.00
R (mV)	1.59±0.10	1.7±0.11	1.47±0.20	1.77±0.17
QT (ms)	0.47±0.01***	0.54±0.02***	0.46±0.02****	0.57±0.02****
T (Neg, Pos, Biphasic)	Neg=4, Biphasic=7	Neg=5, Biphasic=6	Neg=4, Biphasic=7	Neg=5, Biphasic=6
QRS-T angle	6.59±0.85	8.64±1.25	3.60±0.45****	8.18±0.82****

Data represented as mean±SEM,

* p<0.05 compared with control group, **p<0.001 compared with control group,

*** p<0.0001 compared with control group

**Table-3:** Coefficient of determination (R^2^) between base apex and modified precordial lead system.

Variables	Coefficient of determination (R^2^)	

	Base apex	Modified precordial
P (ms)	0.0003	0.0408
P (mV)	0.0216	0.0016
PR (ms)	0.0192	0.0107
QRS (ms)	0.0263	0.2707
R (mV)	0.6691	0.1042
QT (ms)	0.6691	0.5059

**Table-4:** Mean electrical axis parameters in two lead systems.

Lead system	Base apex		Modified precordial	
	
	Control	Athlete	Control	Athlete
Vector	−42.09±49.46	−18.00±51.14	−117.82±3.95	−119.18±4.95
Quadrant	−16.36±56.69	−16.36±56.69	−106.36±4.72	−105.00±4.77

Data represented as mean±SEM

## Discussion

This is the first study to assess the cardiac electrophysiological variables using a new lead system in Thai native crossbred horses. In the present study, the R wave amplitude was lower in the control group compared to the athletic group, which similar to the previous study that the R wave amplitude could be explained for the cardiac adaptation during athletic training [14,15]. The QT segment alterations suggest that the changes in cardiac repolarization and the cause of QT segment changes have not been specifically evaluated in the horse. However, across many other species, it serves as an indicator of life-threatening arrhythmias [16]. There was a very large difference in MEA between the two lead systems, which was similar to the results of the previous studies [17]. However, these results showed that the modified precordial lead system showed minor variation when compared with the results of the base apex lead systems. Echocardiography showed increased left atrium and left ventricular dimensions in athlete group, whereas left ventricular fractional shortening did not differ significantly between the groups. The changes of the left atrium and left ventricular internal diameter are consistent with what has been previously reported in athlete horses [18]. Although there was no significant difference regarding the left ventricular contraction function, stroke volume was increased and heart rate was decreased in athlete group (Table-5). These results might be indicated that exercise training had an influence on heart dimensions and were similar to the finding of a previous study that reported that athletes had larger hearts and larger stroke volumes than non-trained humans [19,20]. However, further study is required for applying the new modified precordial leads system in athletic horses.

**Table-5:** Echocardiographic parameters.

Parameters	Control	Athlete
IVSd (cm)	2.75±0.12	3.02±0.07
LVIDd (cm)	7.57±0.31*	8.50±0.33*
LVPWd (cm)	2.98±0.14	3.29±0.08
IVSs (cm)	3.52±0.1	3.55±0.06
LVIDs (cm)	4.90±0.27	5.65±0.39
LVPWs (cm)	3.51±0.17	3.58±0.07
EF (%)	61.40±2.85	64.00±2.72
FS (%)	34.09±2.07	36.25±1.76
SV (mL)	194.93±17.54*	247.90±14.11*
CO (L/min)	8.11±0.72	8.15±0.66
HR (bpm)	42.64±2.27**	32.90±1.77**
LA/AO ratio	1.19±0.02****	1.26±0.047****

Data represented as mean±SEM,

* p<0.05 compared with control group, \

** p<0.01 compared with control group,

***p<0.001 compared with control group,

**** p<0.0001 compared with control group. IVSd: Diastolic interventricular septum thickness, IVSs: Systolic interventricular septum thickness, LVIDd: Left ventricular end-diastolic diameter, LVIDs: Left ventricular end systolic diameter, LVPWd: Left ventricular wall diastolic thickness, LVPWs: Left ventricular wall systolic thickness. EF: The left ventricular ejection fraction, FS: Fractional shortening, SV: Stroke volume, CO: Cardiac output

## Conclusion

In this study, a new lead system of cardiac electrophysiological evaluation was proposed and successfully applied in measuring the ECG signals in horses. From the experimental results, a modified precordial lead system is suitable for measuring electrocardiographic signal. The proposed system could provide a more reliable method than a base apex lead system and might be useful to apply in the health screening in the future.

## Authors’ Contributions

SP: Principle investigator, drafted and revised the manuscript. WC: A research coordinator, data collection and analysis. KK, NW, NN, WW and SS: Data collection and analysis. All authors read and approved the final manuscript.
